# Biomedical optics applications of advanced lasers and nonlinear optics

**DOI:** 10.1117/1.JBO.25.4.040902

**Published:** 2020-04-23

**Authors:** Christopher B. Marble, Vladislav V. Yakovlev

**Affiliations:** a Texas A&M University, Department of Physics and Astronomy, College Station, Texas, United States; b Texas A&M University, Department of Biomedical Engineering, College Station, Texas, United States

**Keywords:** nonlinear optics, ultraviolet spectroscopy, infrared spectroscopy, terahertz generation

## Abstract

**Significance:** 2019 SPIE Photonics West conference hosted over 5000 presentations. Some important presentations in the Industrial Laser, Laser Source and Application (LASE) and Optoelectronics, Photonic Materials and Devices (OPTO) sections of the SPIE Photonics West conference have a risk of being overlooked by the biomedical community despite their implications for the field of biophotonics. We review some recent advances in the area of development coherent radiation sources in the infrared (IR), ultraviolet (UV), and terahertz (THz) regimes.

**Aim:** Recent advances in coherent radiation sources in the IR, deep UV, and THz regimes were outlined, and the importance of each presentation to one or more promising biomedical applications was assessed.

**Approach:** Presentations and proceedings from the LASE and OPTO sections were reviewed for inclusion. Emphasis was placed on talks from the Nonlinear Frequency Generation and Conversion: Materials and Devices XVIII conference, and the Terahertz, RF, Millimeter, and Submillimeter-Wave Technology and Applications XII conference. Conference sections that directly focused on biomedical applications were excluded.

**Results:** Enhanced IR supercontinuum generation with compact supercontinuum sources may allow for real-time biomarker detection and create new opportunities for imaging tissues using the third biological window (1600 to 1850 nm). Efficient methods to generate deep UV (200 to 260 nm) radiation allow for the study of biologically important molecules through techniques such as resonance Raman spectroscopy while avoiding fluorescence overlap. Likewise, novel and improved THz generation methods seek to bridge the “THz gap” that has previously limited biomedical applications.

**Conclusions:** Advances in coherent radiation sources in the IR, UV, and THz regimes have created new opportunities for biomedical optics research.

## 1 Introduction

This review highlights the presentations in the Industrial Laser, Laser Source and Application (LASE) and Optoelectronics, Photonic Materials and Devices (OPTO) conferences hosted as part of the 2019 SPIE Photonics West symposium, outlines the impact of these advances to the biological sciences, and offers future biological applications for these techniques and materials. The 2019 SPIE Photonics West symposium, held in San Francisco, California, on February 2 to 7, hosted thousands of presentations spanning the fields of biophotonics, laser–tissue interaction, spectroscopy, nonlinear optical effects, laser design, and optoelectronic materials. While the Biophotonics, Biomedical Optics, and Imaging (BiOS) section hosted presentations on biomedical applications of laser technology, the choice of Nobel laureate Dr. Donna Strickland to give a BiOS keynote speech emphasizes the importance of identifying and implementing advancements in laser physics and engineering for biomedical applications. Dr. Strickland was awarded half of the 2018 Nobel Prize in physics with Dr. Gérard Mourou “for their method of generating high-intensity ultrashort optical pulses” through chirped pulse amplification (CPA).^1^^,^^2^ Since its discovery, CPA has become a standard method of generating ultrashort high-intensity laser pulses for biomedical studies.^3^^–^^7^

To further understand the goals of laser technology development, we review and analyze presentations at the SPIE Photonics West conference and connect them to possible biomedical applications. In this attempt to provide an overview and highlight talks from the LASE and OPTO conferences, we focus on talks from conference sections that may have been overlooked by the biomedical community, such as the Nonlinear Frequency Generation and Conversion: Materials and Devices XVIII conference (52 talks, 13 invited talks, and 14 posters) and the Terahertz, RF, Millimeter, and Submillimeter-Wave Technology and Applications XII conference (74 talks, 10 invited talks, and 16 posters). We deliberately avoid LASE/OPTO conferences where talks were directly focused on biomedical applications, such as the Frontiers in Ultrafast Optics: Biomedical, Scientific, and Industrial Applications XIX conference, Microfluidics, BioMEMS, and Medical Microsystems conference, and Adaptive Optics and Wavefront Control for Biological Systems V conference.

## 2 Supercontinuum Generation in the Infrared

Chemical imaging and sensing often relies on hyperspectral imaging in the molecular fingerprint region, which spans through the mid-infrared (IR) part of the spectrum. Supercontinuum generation allows for the production of intense, broadband, and coherent light sources across the near- and mid-IR region. Broadband thermal sources are not coherent, and other methods for generating broadband mid-IR pulses, such as optical parametric amplification and synchrotron radiation, are significantly more expensive and not portable. In this section, we focus on presentations that describe efforts to generate high-power, coherent supercontinuum in the IR by propagating a high-intensity pulse through a nonlinear medium. Presentations are further subgrouped based on a type of a medium (optical fiber versus bulk material).

### 2.1 Fiber Supercontinuum Sources

Dr. Ohishi’s group at Toyota Technological Institute, Japan, reported their efforts in designing a tapered telluride fiber. Their work focused on designing optical fibers that have low and flat dispersion curves throughout the near- to mid-IR region, called an all normal dispersion fiber as these fibers are less likely to suffer from noise due to nonlinear effects in the fiber including source laser instability and soliton fission. The design of the tapered fiber was optimized using computer simulation. The optimized 4-cm-long fiber produced a simulated continuum from ∼1.04 to 4.34  μm by propagating 200-fs, 2.0-μm pulses at an incident pulse power of 44 kW.^8^

Dr. Sergey Mirov (University of Alabama, Birmingham) discussed chromium-doped and iron-doped zinc sulfide (ZnS) and zinc selenide (ZnSe) as mid-IR analogs for Ti:sapphire lasers with chromium dopant used for femtosecond lasing between 2 and 3  μm and iron dopant for 3.5 to 5  μm lasing with the peak powers up to 8 MW for femtosecond pulses. He attributed this to the tetrahedral coordination of the Cr and Fe ions in the II–VI structure resulting in a high absorption cross section and spin-allowed emission in the mid-IR. Using a Cr:ZnS laser, his group generated a low-power continuum from 1.2 to 3.7  μm using a ${\mathrm{Si}}_{3}N_{4}$ (SiN) fiber prepared by Dr. Tobias Kippenberg’s group. The advantage of SiN fibers is that they can be produced by standard CMOS processing unlike other exotic fiber materials.^9^

The fiber sources from Dr. Ohishi’s and Dr. Mirov’s groups are perfectly poised for imaging breast cancer. Supercontinuum fibers sources are used in high-resolution optical coherence tomography systems to provide high-penetration-depth images.^10^^,^^11^ Since the fiber sources from Dr. Ohishi’s and Dr. Mirov’s groups are centered around 2  μm, the sources could be used for imaging breast tissue through the third near-IR window (1600 to 1850 nm). This part of the optical spectrum is relatively unexplored for optical imaging, and, if successful, would result in substantially reduced scattering as compared to imaging using shorter wavelengths in the first near-IR window (650 to 950 nm) and can potentially improve tumor imaging.^12^^,^^13^

Dr. Mohammed Islam, from the University of Michigan and Omni Sciences Inc., gave an invited talk on applying existing commercial telecom equipment to generate supercontinuum from 1.57 to 12  μm. Unlike other systems where only one fiber was used, the broadband continuum was achieved by cascading a fused silica fiber, a ZBLAN fluoride fiber, a sulfide fiber, and a high-numerical-aperture selenide fiber. By cascading the fibers, higher output powers and efficiencies were achieved compared to generating mid-IR continuum directly with the sulfide or selenide fibers.^14^

The chemical composition of gases can be determined by measuring the wavelength-dependent absorption with a broadband supercontinuum (supercontinuum absorption spectroscopy).^15^ This technique could be applied to measuring biomarkers in human breath such as NO, CO, volatile organic compounds, including ethanol, methane, and acetone, as well as sulfur- and nitrogen-containing compounds.^16^ A compact, broadband continuum source as the one developed by Dr. Islam could provide the opportunity of real-time detection and analysis of biomarkers unlike gas chromatography or mass spectrometry which requires breath samples to be collected and sent to the laboratory for analysis.^17^

### 2.2 ZnS and ZnSe Materials as Continuum Sources

An alternative approach to supercontinuum generation is to generate the continuum using ZnSe or ZnS plates. Several recent publications have explored ZnS’s and ZnSe’s unusually strong nonlinear properties which allow it to undergo extremely efficient frequency conversion through harmonic generation and continuum broadening over a range of wavelengths spanning the mid-IR.^18^^–^^21^ Using existing polycrystalline ZnSe plates, broadband continuum can be generated using material thicknesses as small as 3 to 10 mm.^18^^–^^21^ A collaboration between the U.S. Army Research Laboratory, Ohio State University, the University of Arizona, and SURVICE Engineering Co. reported measurements of the nonlinearity of polycrystalline ZnSe $\left[n_{2}=(1.2\pm 0.3)\cdot 10^{-14}\;{\mathrm{cm}}^{2}/W\right]$ and ZnS $\left[n_{2}=(5.0\pm 0.3)\cdot 10^{-15}\;{\mathrm{cm}}^{2}/W\right]$ at 3.9  μm and demonstrated that simulations of the unidirectional pulse propagation equation with incorporated ionization effects could explain the broadband continuum generation experimentally observed in these media.^22^

While current studies have focused on polycrystalline ZnSe, Dr. Peter Schunemann (BAE Systems) detailed efforts to grow large single ZnSe and ZnS crystals through physical vapor transport and chemical vapor transport (CVT). Dr. Schunemann reported that CVT with iodine resulted in the successful growth of single crystals up to $1\;{\mathrm{cm}}^{3}$ in size, and those crystals could be doped with chromium. The aim of Dr. Schunemann’s and Dr. Kawilski’s work was to achieve greater phase matching by building ZnSe windows with orientation-patterned single-crystal ZnSe layers. Due to the low dispersion of ZnSe, the grating periods can be large, and rather broadband pump pulses can be used to achieve frequency conversion. The enhanced phase matching leads to a potentially greater efficiency of harmonic and continuum generation, which in turn would allow for higher efficiency mid-IR continuum generation.^23^

Similar to fiber-based light sources, supercontinuum generated by ZnSe windows can be applied to a wide array of applications ranging from hyperspectral imaging^13^ to cancer screening.^12^ Compared to fiber sources, polycrystalline ZnS and ZnSe windows are relatively inexpensive, compact, and do not come with the complications of coupling a cascade of fibers. ZnS and ZnSe windows may play a key role in future miniaturized mid-IR continuum sources.^23^ Furthermore, developing CVT methods to dope ZnS and ZnSe with chromium is of interest for designing IR lasers as emphasized in Dr. Mirov’s talk.^9^

### 2.3 Near-Infrared Continuum Generation as a Retinal Hazard

Our group presented results on simulated continuum generation in water and eye tissues. In the talk, previous observations of continuum generation in a cuvette of water from 1200 to 1400 nm were compared to the results of an electric field propagating split-step simulation. The importance of incorporating self-focusing and accounting for possible plasma effects for simulating pulse propagation in water were emphasized. The end of the talk focused on simulating femtosecond pulses under the current American National Standards Institute maximum permissible exposure limit in a reduced human eye model. These simulations raised concerns of possible retinal hazards for pulses between 1350 and 1400 nm with a pulse duration of around 100 fs due to a combination of the initial pulse’s transform limited bandwidth and continuum broadening.^24^ Despite numerous studies of retinal hazards and eye safety including many published in the *Journal of Biomedical Optics*,^25^^–^^30^ retinal hazards from nonlinear effects including continuum broadening remains relatively unexplored and open to further experimental and simulation studies.^31^^–^^36^

## 3 Advances in UV Generation

While numerous SPIE talks discussed IR radiation sources, advances in ultraviolet (UV) radiation sources were also discussed. Milliwatt-level UV sources have been developed by utilizing ion lasers, UV diodes, and by nonlinear frequency conversion of the near-IR lasers. Each method has its own limitations. Ion lasers can generate Watts of power, but usually operate as continuous-wave (CW) sources at discrete wavelengths. Conventional UV diode laser systems suffer from limitations in power and spatial mode quality, while nonlinear conversion from the near-IR laser sources in bulk media is very inefficient for CW operation and often hard to maintain for day-to-day operations.^37^

Aller et al. (AdvR, Inc.) proposed a new device based on a two-part magnesium oxide-doped lithium niobate (MgO:LM) waveguide to enhance nonlinear optical conversion efficiency from the near-IR to the UV. The first section of the waveguide was periodically poled to optimize for second-harmonic generation from 1064 to 532 nm, and the second was adjusted for sum frequency generation of the fundamental and second harmonic to generate UV light at the 355-nm wavelength. The optimized waveguide, fabricated by AdvR, Inc., converted 200 mW of the IR power at the 1064-nm wavelength to 3 mW of the UV power. The lifetime was evaluated, and continuous nondiminishing performance was demonstrated for thousands of hours.^37^

For deep-UV generation (200 to 266 nm), there is a distinct lack of commercially available crystals for frequency conversion compared to beta barium borate or lithium triborate/cesium lithium borate crystals (for 266 and 355 nm, respectively). Borate crystals have many of the properties required for deep-UV frequency conversion including an acentric structure, low-UV absorption, reasonable nonlinear coefficient, and a moderate birefringence. The lack of commercial success with borate is, in part, due to issues related to its hygroscopicity and the lack of phase matching for the direct second-harmonic generation for shorter wavelengths. Alternatives to borate crystals were known, but commercial fabrication remained elusive. At SPIE, Dr. Kolis (Clemson University) presented large ${\mathrm{Sr}}_{2}{\mathrm{Be}}_{2}B_{2}O_{7}$ crystals with ordered P-6 structure, which could be achieved with hydrothermal growth, a substantial improvement to previously reported, disordered crystals grown in fluxes. He also discussed their recent successes in large single-crystal fabrication for ${\mathrm{KBe}2\mathrm{BO}}_{3}F_{2}$ (KBBF) and ${\mathrm{RbBe}}_{2}{\mathrm{BO}}_{3}F_{2}$ (RBBF). Using KBBF and RBBF, they report generation of 1- and 10-mW UV pulses (picosecond- and femtosecond-order pulse duration) for wavelengths ranging from 177 to 235 nm and were able to take angle-resolved photoemission spectroscopy measurements using the generated UV light. The growth of large single crystals for deep-UV frequency conversion and attempts to commercialize it with companies such as HT Crystal Solutions will facilitate further research in biospectroscopy using UV radiation.^38^

Unlike IR radiation, which is mainly absorbed by water in tissues, deep-UV radiation (200 to 260 nm) is preferentially absorbed by amino acids, proteins, and DNA leading to numerous biological applications.^38^^,^^39^ Since the epidermis absorbs UV radiation, UV spectroscopy for human tissues has been limited *in vivo* to imaging of the human epidermis.^40^^,^^41^ Other applications include standoff detection, high-resolution microsurgery on the retina, nanostructure fabrication, and deep-UV Raman spectroscopy.^38^

The deep-UV Raman signal is several orders of magnitude stronger than visible Raman signal due to a combination of shorter wavelength (roughly 2 orders of magnitude scattering cross-section enhancement) and electronic resonant enhancement with many biological molecules (4 to 6 orders of magnitude signal increase). As a bonus, the fluorescent emission spectrum for biomolecules usually begins at 265 nm or longer wavelengths; hence, there is no significant spectral overlap between fluorescent and Raman signals for the UV-Raman measurements. At the conference, our research group discussed UV-Raman spectroscopy as one of the potential applications for deep-UV radiation sources and proposed several methods to generate sufficient UV light for Raman measurements. The first method used the fourth-harmonic generation of the high repetition rate Nd:YAG laser operating at 946 nm, which has an obvious advantage of simplicity but lacks tunability.^42^ Discrete tunability was achieved by propagating 1064-nm light through ${\mathrm{YVO}}_{4}$ seeded with white light continuum from a fiber. The resulting first and second Raman lines were wavemixed with 1064 nm to achieve multiple fixed UV frequencies.^43^ Finally, a broadband tunability with high stability and narrow bandwidth was achieved through a CW seeded optical parametric amplifier.^44^

The talk highlighted UV-Raman applications, including proton conformational changes, detecting agricultural runoff, and pesticides in environmental samples.^45^ UV resonance Raman has also been applied *ex vivo* to study human bone fragility, protein structure, aging of DNA through telomere length, and conformation of DNA in a nucleus.^46^^,^^47^

## 4 Terahertz Generation and Spectroscopy

Terahertz (THz) science has been limited for decades by a lack of sufficiently intense THz sources, the so-called “THz gap.” The two most common approaches to generate THz radiation utilize photoconductive antenna (PCA) or optical rectification (OR) with less common methods based on difference frequency generation, quantum cascade lasers, and p-Ge lasers.^48^

Several presentations discussed methods to improve OR and PCA techniques. Dr. Ian McNee’s (Microtech Instruments) work with Dr. Andrea Markelz’s group at the University of Buffalo and Dr. Peter Schunemann at BAE Systems focused on improving existing OR techniques. In Dr. McNee’s talk, he reported generating 0.9 to 3.8 THz pulses with average power up to 15  μW by focusing 180-fs, 1064-nm pulses from an 8-W fiber laser into orientation patterned gallium phosphide (OP-GaP). To tune the frequency of THz pulses, OP-GaP crystals with different periods were employed. They demonstrated this system could perform anisotropic THz spectroscopy by detecting the change in the THz transmission in the time domain as the orientation of a sucrose crystal was rotated with respect to the linearly polarized THz pulses.^49^

Likewise, Dr. Kuwashima (Fukui University of Technology) presented work on a PCA system done in collaboration with researchers at the University of Fukui, Japan Coast Guard Academy, and Osaka University. They reported their success in efficiently generating a stable THz waves from 0.1 to 1.0 THz using a PCA excited using chaotic oscillation in a CW multimode diode laser. Using a CW diode laser, compared to a Ti:sapphire laser, they generate THz waves with PCA at a greatly reduced total cost.^50^

Other talks focused on novel THz designs such as the ultrathin Fe/Pt spintronic emitters presented by Dr. Beigang’s group (Technical University of Kaiserslautern, Germany). Dr. Beigang’s group reported that spintronic emitters made by electron-beam evaporation of nanometer thickness Fe or Pt onto a thick MgO substrate were able to generate THz radiation at pump frequencies of 400, 800, and 1550 nm with comparable efficiencies. By operating at 1550 nm, the system can be pumped using a cheaper and more compact fiber laser system.^51^

Increasing the maximum power, while reducing the cost, complexity, and size of THz systems, is essential for future applications. Despite a relatively high cost and existing limitations, THz spectroscopy has already been explored in biomedical spectroscopy and imaging. Similar to UV spectroscopy, the THz radiation is strongly absorbed in the skin with applications limited to surface and near-surface imaging including burn characterization,^52^^,^^53^ corneal health,^54^^,^^55^ early detection of skin cancer,^52^ and dental caries.^56^ Relaxing the *in vivo* restriction, THz spectroscopy has been applied to study excised tissues including cancer of the brain,^57^ breast,^52^ and colon,^58^ cirrhosis of the liver,^52^ traumatic brain injury,^59^ and Alzheimer’s disease.^60^

Many THz applications rely on time-domain spectroscopy using femtosecond to picosecond pulses and are limited by the refresh rate of the system. At the conference, Dr. Christoph Lange and Dr. Rupert Huber’s group at the University of Regensburg presented an all-optical approach using a field programmable gate array and an acousto-optical delay line, which allows for THz time-domain spectroscopy scan rates up to 36 kHz. Other all optical time-domain spectroscopy methods have achieved kilohertz repetition rates, but with limitations such as dead time (asynchronous optical sampling) or complex cavity elements (optical sampling through cavity tuning).^61^^,^^62^ By providing kilohertz repetition rates without dead time, THz time-domain spectroscopy can be applied to real-time imaging of living biological specimens, rapid chemical analysis, and studies of nonrepeatable biological processes.

For some applications where refresh rates of 0.1 to 120 Hz with limited bandwidth or resolution are acceptable, frequency-domain spectroscopy can serve as a valid alternative to time-domain spectroscopy. Frequency-domain spectroscopy, sometimes called CW spectroscopy, has gained attention as a more compact, lower cost alternative to time-domain spectroscopy. Unlike time-domain spectroscopy, frequency-domain spectroscopy systems utilize photomixing of two CW sources to generate THz waves. The scan speed of frequency-domain systems is limited by the tuning speed of the two-laser system; however, recent efforts reported by Dr. Lars Liebermeister’s group (Fraunhofer Heinrich-Hertz-Institute) at Photonics West demonstrate that frequency-domain spectroscopy can be performed at refresh rates up to 120 Hz at a cost of bandwidth (200 GHz) and resolution (800 MHz). Furthermore, in principle, the detection system presented could be miniaturized onto a single photonic chip as it relies on semiconductor lasers and has no moving parts or reliance on free space optics.^63^

## 5 Summary and Outlook

The annual SPIE Photonics West conference provides a forum for communicating advances in laser design and applications. The 2019 Photonics West conference sections on advances in radiation sources showcased improved methods to generate supercontinuum in the IR, deep-UV radiation, and THz radiation to the biomedical research community for spectroscopic applications. These advances included reductions in cost, size, and complexity of these devices, which is critical in bringing these sources out of specialized labs and making them more available for general scientific research. Each of the techniques and methods discussed in this paper has limitations and advantages that should be considered in detail when choosing whether to apply it to a particular application. In this proceeding, we sought to provide a broad overview of new methods or improvements to existing techniques without dwelling in depth on a specific application. We recommend the reader seek more specialized reviews for an in-depth discussion of a particular topic of interest.
